# TB/FLU-06E Influenza Vector-Based Vaccine in the Complex Therapy of Drug-Susceptible and Drug-Resistant Experimental Tuberculosis

**DOI:** 10.3390/pharmaceutics16070857

**Published:** 2024-06-25

**Authors:** Anna-Polina S. Shurygina, Natalia V. Zabolotnykh, Tatiana I. Vinogradova, Maria L. Vitovskaya, Marine Z. Dogonadze, Kirill A. Vasilyev, Zhanna V. Buzitskaya, Petr K. Yablonskiy, Dmitriy A. Lioznov, Marina A. Stukova

**Affiliations:** 1 Smorodintsev Research Institute of Influenza, The Ministry of Health of the Russian Federation, 197022 Saint-Petersburg, Russia; 2 Saint-Petersburg State Research Institute of Phthisiopulmonology, The Ministry of Health of the Russian Federation, 194064 Saint-Petersburg, Russia

**Keywords:** TB-specific immunotherapy, mucosal vaccine, drug-resistant tuberculosis, influenza vector, ESAT6

## Abstract

The steady rise of drug-resistant tuberculosis (TB), which renders standard therapy regimens ineffective, necessitates the development of innovative treatment approaches. Immunotherapeutic vaccines have the potential to effectively regulate the anti-TB immune response and enhance the efficacy of anti-TB treatment. In the present study, we aimed to evaluate the potency of the mucosal vector vaccine TB/FLU-06E as part of a complex treatment regimen for drug-susceptible (DS) or drug-resistant (DR) tuberculosis in C57BL/6 mice. Incorporating TB/FLU-06E into the treatment protocol significantly increased the effectiveness of therapy for both forms of tuberculosis. It was evidenced by higher survival rates and reduced pulmonary bacterial load (1.83 lg CFU for DS tuberculosis and 0.93 lg CFU for DR tuberculosis). Furthermore, the treatment reduced pathomorphological lesions in the lungs and stimulated the local and systemic T-helper 1 (Th1) and cytotoxic T-lymphocyte (CTL) anti-TB immune responses. Thus, therapeutic immunization with the TB/FLU-06E vaccine significantly enhances the efficacy of tuberculosis treatment, which is particularly important in DR tuberculosis.

## 1. Introduction

The marked increase in multidrug-resistant and extensively drug-resistant (MDR/XDR) tuberculosis highlights the urgent need to address these forms of infection spreading even in developed countries [1,2]. Treating MDR/XDR tuberculosis is complex and expensive due to the common resistance to the most important and effective anti-TB drugs [3]. Drug-resistant tuberculosis often progresses to chronic forms with the persistence of non-replicating mycobacteria, inevitably resulting in a relapse of infection [4,5]. Immune response disorders in DR tuberculosis are caused both by the induction of T-cell dysfunction by persistent mycobacteria, leading to a prolonged course and chronicity of the tuberculosis process [6], and by the immunosuppressive effect of anti-TB drugs during intensive and prolonged polychemotherapy, which is necessary for treating MDR tuberculosis [7]. A way to overcome this problem is to stimulate the macroorganism’s immune system by immunotherapeutic agents. They can promote stable functional Thl cell activity to ensure effective immune control over *Mycobacterium tuberculosis* (*M. tuberculosis*) replication, as well as to balance the depressive effects of anti-TB drugs on cellular immunity [7,8,9,10]. Immunotherapy is particularly relevant in DR tuberculosis because existing anti-TB drugs target only replicating pathogens, and the persistence of mycobacteria, combined with the prolonged course and chronicization of the tuberculosis process, causes a pronounced imbalance of the immune system [6].

In recent years, in addition to investigating their efficacy in preventive vaccination, anti-TB vaccine candidates have been studied as part of therapeutic immunization regimens [4]. Specific therapeutic immunization has been shown to reduce the duration of causal therapy in experimental DS tuberculosis [11]. It can also provide accelerated immune protection if the organism repeatedly encounters *M. tuberculosis*, which is crucial for preventing tuberculosis reactivation [12,13].

Here we used TB/FLU-06E, a vaccine based on an attenuated influenza vector expressing the mycobacterial antigen ESAT-6 (early secreted antigenic target 6 kDa) [14,15], as an immunotherapeutic agent. ESAT-6 is known to be an immunodominant *M. tuberculosis* antigen with strong immunomodulatory effects [16,17]. Vaccines based on ESAT-6 have demonstrated a high protective potential in preventive, post-exposure, and therapeutic *M. tuberculosis* immunization [18,19,20,21,22]. Furthermore, the TB/FLU-06E vaccine’s immunomodulatory capacity is largely due to the properties of the influenza vector itself, which is based on influenza A virus strains with a modified nonstructural (NS) genome. Influenza virus carrying mutations in the NS gene in the viral replication zone can induce the production of a wide range of pro-inflammatory cytokines, thus promoting a comprehensive systemic immune response with Th1 polarization and CD8+ T-cell activation [23,24]. 

This study aimed to investigate the efficacy of the protective TB/FLU-06E vaccine, which activates T-cellular immunity to prevent infection, for specific immunotherapy of experimental tuberculosis caused by mycobacteria with different drug susceptibilities.

## 2. Materials and Methods

### 2.1. TB/FLU-06E Production

Chimeric influenza virus A/Guangdong-Moonan/SWL1536/2019A_NS_124__ESAT-6 (TB/FLU-06E) was generated by reverse genetics as described earlier [14]. The vaccine candidate TB/FLU-06E was produced in developing chicken embryos. The harvest was purified by consequent clarification, concentration, and diafiltration steps and formulated in a sucrose-phosphate-glutamate stabilizing buffer (SPGN). The stabilizing buffer was given as a “mock” control in animal studies.

### 2.2. Laboratory Animals

Specific-pathogen-free C57BL/6 mice, 6–8 weeks old, were purchased from the Nursery for Laboratory Animals Pushchino (Shemyakin and Ovchinnikov Institute of Bioorganic Chemistry RAS, Moscow, Russia). All animal studies followed the international recommendations (Directive 2010/63/EU) and the protocols approved by the Bioethics Committee of the State Research Institute of Phthisiopulmonology.

### 2.3. Experimental Design

Drug-susceptible tuberculosis in C57BL/6 mice (n = 73, Table S1) was modeled by inoculation of the lateral tail vein with a virulent *M. tuberculosis* H37Rv strain, 6.0 lg CFU/animal suspended in 200 µL DPBS. Drug-resistant tuberculosis (n = 73, Table S2) was induced by inoculation of the same dose of the clinical strain 558 of *M. tuberculosis* Beijing genotype, resistant to four anti-TB drugs: isoniazid, rifampicin, streptomycin, and pyrazinamide. Both strains were obtained from the collection of Saint-Petersburg State Research Institute of Phthisiopulmonology. In each experiment, infected animals were selectively examined for multiple submiliary or single miliary foci of specific inflammation in the lungs. Therapy was initiated if this post-mortem examination confirmed pathological changes in the lungs and was administered based on the profile of tuberculosis drug sensitivity. Anti-TB therapy for drug-susceptible tuberculosis included 10 mg/kg of isoniazid (H) and 10 mg/kg of rifampicin (R); for drug-resistant tuberculosis, 30 mg/kg of amikacin (A), 20 mg/kg of ethambutol (E), 14 mg/kg of bedaquiline (Bq), and 12 mg/kg thioureidoiminomethylpyridinium perchlorate (perchlozone, Tpp) was used.

Vaccination with TB/FLU-06E was performed intranasally (6.0 lg EID_50_/30 µL/animal), following different schedules in each experiment. Untreated infected mice and mice receiving anti-TB drugs only (control therapy) served as vaccine therapy controls. After 2.5 months (DS) or 4 months (DR) of causal therapy, the efficacy of the vaccine therapy was assessed through survival rates, macroscopic evaluation of lung lesions, isolation of *M. tuberculosis* from the lungs, and lung histopathology. The systemic TB-specific T-cell immune response was measured by intracellular cytokine staining (ICS) of splenocytes. The gaiting strategy and representative plots are shown in Supplementary Materials (Figures S1–S5).

### 2.4. Mycobacterial Load

To quantify the live mycobacteria load, the tissue homogenates were titrated and cultured on a Lowenstein–Jensen solid medium. Bacterial colonies were counted after a 3-week incubation at 37 °C. Titers were expressed as log10 of the mean colony forming units (lg CFU) per lung weight. The detection limit was set to 2 × 10^3^ CFU. A decrease in bacterial load of more than 0.5 lg CFU in comparison with the control groups was considered a positive protective effect.

### 2.5. Histopathology

Lung tissues were fixed in 10% formalin (pH 7.0) and embedded in paraffin. For histopathological studies, 3–4 µm sections were stained with hematoxylin and eosin. Images were captured using the Olympus BX45 microscope (Olympus Corp., Tokyo, Japan) with a camera and the Olympus DP-Soft 5.0 software package (Olympus Corp., Tokyo, Japan).

### 2.6. Intracellular Cytokine Staining

For intracellular cytokine staining (ICS), single-cell suspensions were prepared from spleens. Mechanically dissociated tissues were passaged through a 70 μm cell strainer into RPMI 1640 medium (Gibco, Waltham, MA, USA) supplemented with 10% *v*/*v* FBS (Gibco, Waltham, MA, USA) and 1% penicillin-streptomycin solution (Gibco, Waltham, MA, USA). The cells were washed before and after erythrocyte lysis using an ammonium chloride lysing solution (0.15 M NH_4_Cl, 10 mM NaHCO_3_, 1 mM Na_2_EDTA) and seeded at a density of 1 × 10^6^ cells per well into flat-bottom 96-well tissue culture plates (Nunc, Roskilde, Denmark). The cells were stimulated with BCG (5 μg/mL) in the presence of anti-CD28 antibodies (BioLegend, San Diego, CA, USA) at 37 °C, 5% CO_2_, for 24 h. Medium alone and PMA plus Ionomycin (both Sigma, Saint Louis, MO, USA) were used as negative and positive controls, respectively. Next, the GolgiPlug reagent (BD Biosciences, San Jose, CA, USA) was added, and the cells were cultured for another 6 h. Following incubation, the cells were washed (500× *g*, 5 min) and stained with ZombiRed (BioLegend, San Diego, CA, USA) and surface markers CD8-PECy7 (BioLegend, San Diego, CA, USA), CD4 -PerCPCy5.5 (BD Biosciences, USA), CD 44-BV510 (BioLegend, San Diego, CA, USA), and CD62L-APCCy7 (BioLegend, San Diego, CA, USA). To reduce unspecific cell staining, the TrueStain reagent (BioLegend, San Diego, CA, USA) was used. Subsequently, the cells were washed, and the intracellular staining with IFN-γ-FITC, IL-2-PE, and TNF-α-BV421 (BioLegend, San Diego, CA, USA) was performed using the BD Biosciences Cytofix/Cytoperm kit (BD Biosciences, San Jose, CA, USA) according to the manufacturer’s instructions. Data were collected on the Cytoflex flow cytometer (Beckman Coulter, Bray, CA, USA). The results were analyzed using the Kaluza Analysis 2.2 program (Beckman Coulter, Bray, CA, USA).

### 2.7. Statistical Analyses

The GraphPad Prizm 10.0 software (GraphPad Software, Inc., La Jolla, CA, USA) was employed for statistical analyses. All values were expressed as the mean ± standard deviation (SD) or standard error of the mean (SEM), as indicated. Groups were compared using one-way or two-way ANOVA, followed by Tukey’s multiple comparison test. The significance of the differences between survival rates was evaluated using the log-rank test. Fisher’s exact test was used to analyze the histological data. *p* < 0.05 was considered statistically significant.

## 3. Results

### 3.1. TB/FLU-06E Vaccine Efficacy in the Complex Therapy of Drug-Susceptible Tuberculosis

**Figure 1:**
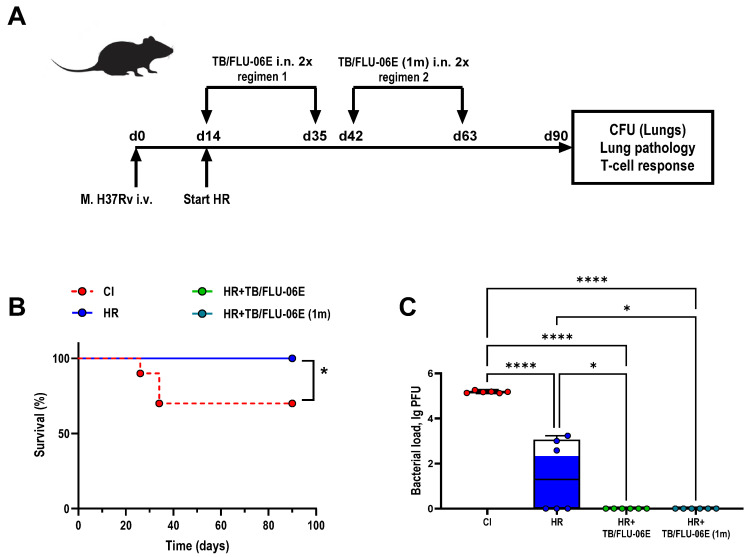
TB/FLU-06E therapy in a drug-suspectable tuberculosis model. (**A**) Design of the experiment. Male 6–8 weeks old C57BL/6 mice were i.v. infected with *M. tuberculosis* H37Rv strain 6.0 lg CFU/animal suspended in 200 µL DPBS. Anti-tuberculosis therapy with isoniazid 10 mg/kg (H) plus rifampicin 10 mg/kg (R) was started after infected animals showed signs of infection (day 14). Therapeutic vaccination was administered in two regimens. Regimen 1: HR+TB/FLU-06E, double vaccination with a 3-week interval, where the first vaccination was performed simultaneously with the start of HR (day 14); Regimen 2: HR+TB/FLU-06E (1m), double vaccination with a 3-week interval, where the first vaccination was performed one month after the start of HR (day 42). (**B**) Survival rates. The significance of differences between groups (n = 10 per group) was calculated using the log-rank test (* *p* = 0.0163). (**C**) *M. tuberculosis* H37Rv loads in the lungs after 2.5 months of therapy (day 90). The data were considered statistically significant when *p* < 0.05, as determined by one-way ANOVA with Tukey’s multiple comparisons test (* *p* < 0.05, **** *p* < 0.0001).

To evaluate the potential of TB/FLU-06E in improving the efficacy of anti-TB therapy in drug-susceptible tuberculosis, C57BL/6 mice were infected with the *M. tuberculosis* H37Rv strain and treated with the first-line antituberculosis drugs (isoniazid 10 mg/kg, subcutaneous (H), and rifampicin 10 mg/kg, intragastric (R)) in average therapeutic doses. The TB/FLU-06E immunotherapy was administered twice (3 weeks apart) following two regimens: at the start of therapy (HR+TB/FLU-06E) and one month after the beginning of HR administration (TB/FLU-06E (m1)) (Figure 1A). In 2.5 months, the activity of effector memory T cells (Tem) was compared with the efficacy of the treatment.

**Figure 2:**
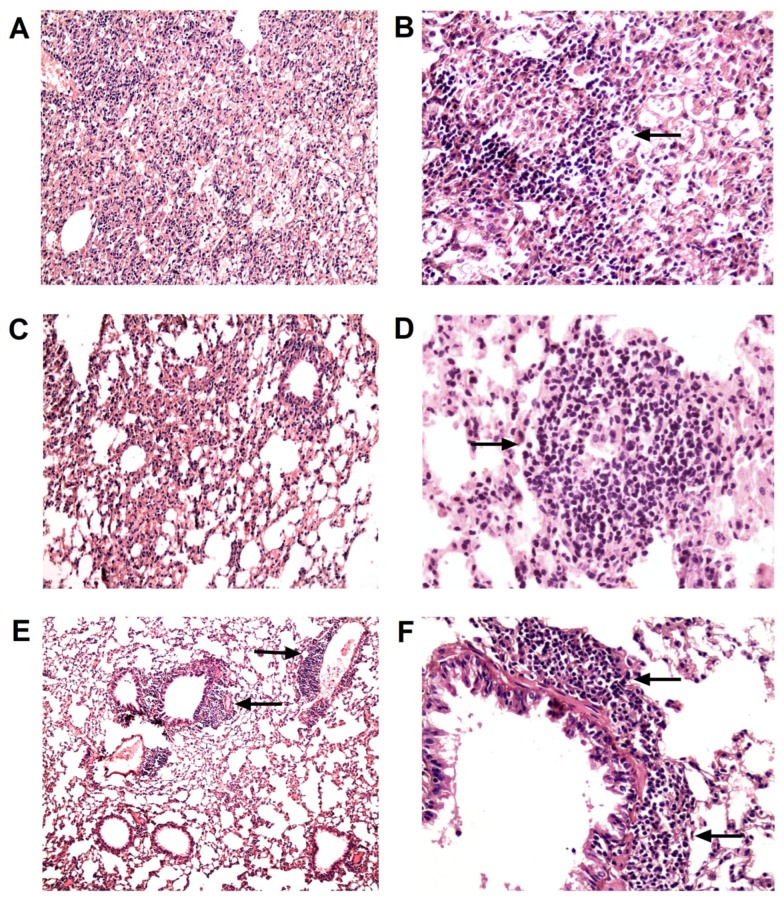
Representative micrographs of histological sections of the lungs from *M. tuberculosis* H37Rv-infected mice 2.5 months after the start of therapy. Large confluent foci of specific infiltration without clear spatial orientation of cells (**A**) and iBALT clusters with a high density of epithelioid cells (**B**) in the lungs of untreated infected mice. Small areas of infiltration (**C**) and a large iBALT cluster with few epithelioid cells (**D**) in the lungs of HR-treated mice. Thickening of interalveolar septa, marked lymphohistiocytic infiltration (**E**), and lymphoid peribronchial iBALT clusters (**F**) in the lungs of HR+ TB/FLU-06E-treated mice. Hematoxylin and eosin staining. Magnification ×300 (**A**,**C**,**E**) or ×600 (**B**,**D**,**F**). The black arrows indicate the described histological changes.

None of the HR-treated animals had died by the end of the experiment, whereas the survival rate in the untreated control group (CI) was 70% (Figure 1B). The lungs of all untreated infected mice (n = 6) showed *M. tuberculosis* growth averaging 5.18 lg CFU (Figure 1C). The lung histology in this group was characterized by the presence of large confluent foci of specific infiltration with no clear spatial orientation of cells and occasionally showed iBALT aggregates with large inclusions of epithelioid cells (Figure 2A,B).

The therapy with anti-TB drugs alone (HR) decreased bacterial growth by 3.38 lg CFU (*p* < 0.0001; Figure 1C). It also reduced lung lesions to isolated small foci of infiltration with an area of two to six alveoli, while epithelioid cell clusters in iBALT interstitial conglomerates decreased in size, being replaced by large periarterial lymphohistiocytic infiltrates in 50% of cases (Figure 2C,D).

Initiating immunotherapy with TB/FLU-06E at the onset of causal treatment (HR + TB/FLU-06E) further reduced the bacterial load by 1.8 lg CFU (HR + TB/FLU-06E vs. HR *p* = 0.0247). As a result, *M. tuberculosis* growth in the lungs was below the detection limit in all animals in this group (Figure 1C). Histological examination of the lungs revealed a significant reduction in specific infiltration foci (*p* < 0.001) and iBALT clusters containing epithelioid cells (*p* < 0. 0005), as well as an increased formation of iBALT lymphoid aggregates (*p* < 0.0005) coupled with perivascular (*p* < 0.02) and peribronchial (*p* < 0.001) lymphohistiocytic infiltration (Figure 2E,F). When TB/FLU-06E was administered one month after the start of HR therapy, its effect on *M. tuberculosis* bacterial clearance from the lungs was the same (HR + TB/FLU-06E (1) vs. HR *p* = 0.0247). However, the reduction in specific lung lesions and the effect on the lymphoid inflammation component were less pronounced.

**Figure 3:**
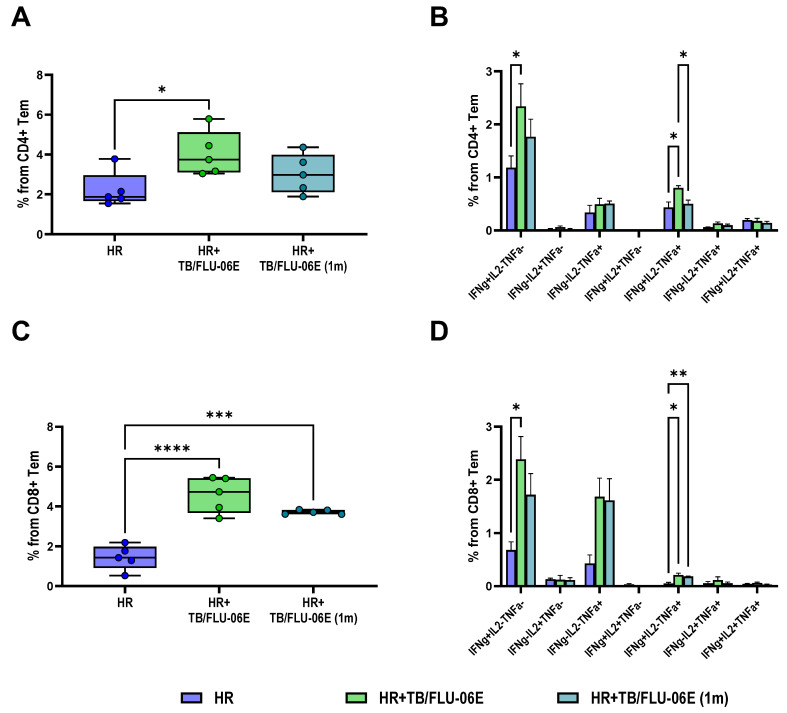
Systemic TB-specific immune response following DS-TB treatment. Proportions of cytokine-producing CD4+ (**A**,**B**) and CD8+ (**C**,**D**) effector memory T cells in spleens of C57BL/6 mice infected with *M. tuberculosis* H37Rv, after 2.5 months of HR therapy with TB/FLU-06E (intranasally, twice). The frequencies of IFN-γ-, TNF-α-, and IL-2-producing CD4+ and CD8+ Tem cells were measured by flow cytometry (ICS). The background signal from cells stimulated only with the medium has been subtracted. The data were considered statistically significant when *p* < 0.05, as determined by one-way or two-way ANOVA with Tukey’s multiple comparisons test (* *p* < 0.05, ** *p* < 0.01, *** *p* < 0.001, **** *p* < 0.0001).

Improvement in the clinical course of DS tuberculosis due to the TB/FLU-06E immunotherapy was accompanied by enhanced activity of CD4+ and CD8+ Tem cells. The TB-specific response in the groups treated with TB/FLU-06E was characterized by an intensive formation of IFN-γ and IFN-γ/TNF-α producing cells (Figure 3).

### 3.2. TB/FLU-06E Vaccine Efficacy in the Complex Therapy of Drug-Resistant Tuberculosis

**Figure 4:**
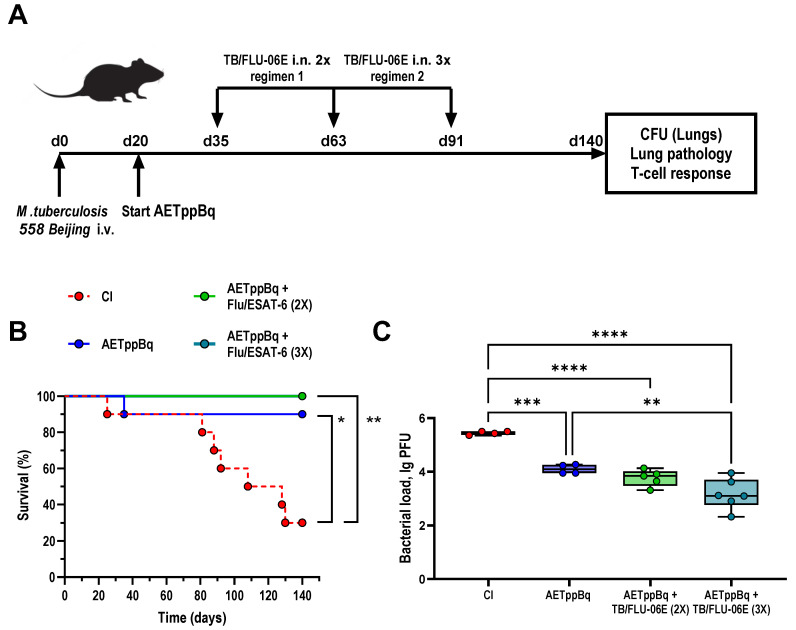
TB/FLU-06E therapy in a drug-resistant tuberculosis model. (**A**) Design of the experiment. Male 6–8 weeks old C57BL/6 mice were i.v. infected with a clinical strain 558 of *M. tuberculosis* genotype *Beijing* 6.0 lg CFU/animal suspended in 200 µL DPBS. Anti-tuberculosis therapy with amikacin 30 mg/kg (**A**), ethambutol 20 mg/kg (E), bedaquiline 14 mg/kg (Bq), and thioureidoiminomethylpyridinium perchlorate 12 mg/kg (perchlozone, Tpp) started after infected animals showed signs of infection (day 20). Therapeutic vaccination was administered in two regimens. Regimen 1: AETppBq + TB/FLU-06E (2×), double vaccination with a 4-week interval, where the first vaccination was performed 2 weeks after the start of AETppBq (day 35); Regimen 2: TB/FLU-06E (3×), triple vaccination 4-week intervals, where vaccination was performed 2 weeks after the start of AETppBq (day 35). (**B**) Survival rates. The significance of differences between groups (n = 10 per group) was calculated using the log-rank test (* *p* = 0.0105; ** *p* = 0.0011). (**C**) *M. tuberculosis* clinical strain 558 Beijing loads in the lungs after 4 months of therapy (day 140). The data were considered statistically significant when *p* < 0.05, as determined by one-way ANOVA with Tukey’s multiple comparisons test (** *p* < 0.01, *** *p* < 0.001, **** *p* < 0.0001).

In mice with drug-resistant TB, the efficacy of TB/FLU-06E immunotherapy was evaluated 4 months after the initiation of therapy (Figure 4A). In this model, C57BL/6 mice were infected with a clinical strain of *M. tuberculosis* of the Beijing family known to be resistant to isoniazid, rifampicin, streptomycin, and pyrazinamide.

**Figure 5:**
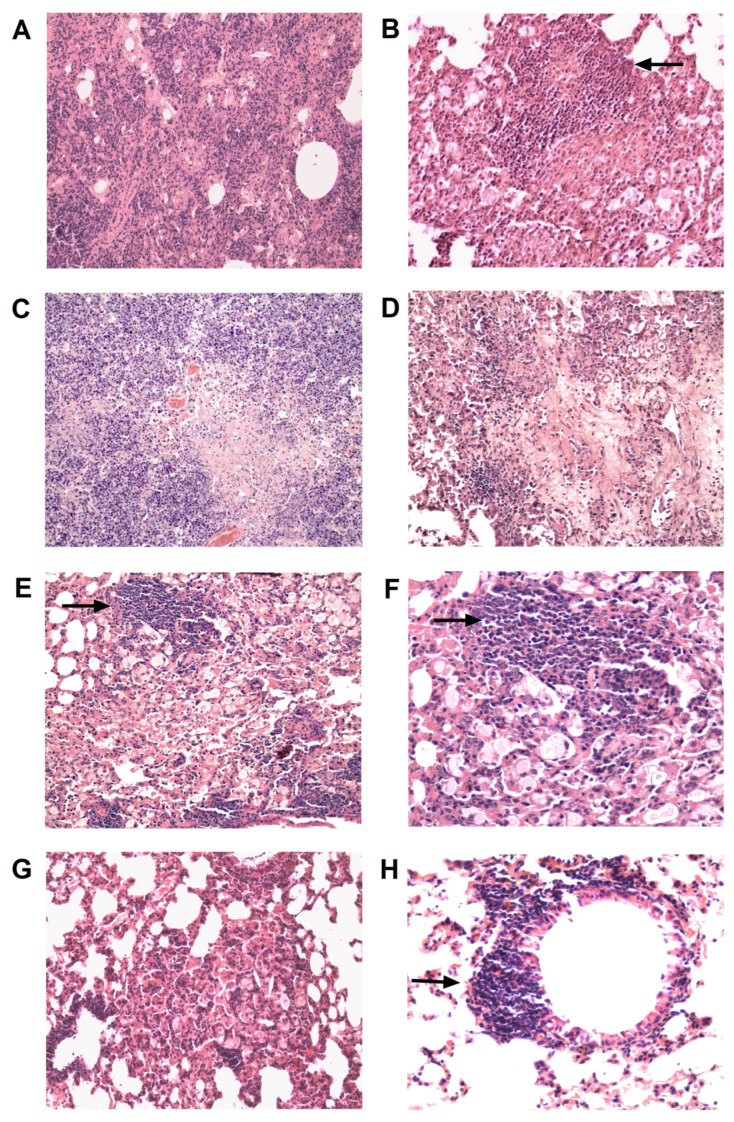
Representative micrographs of histological lung sections from C57BL/6 mice infected with a drug-resistant strain of *M. tuberculosis* (Beijing family) at day 140 post-infection. Large confluent foci of specific infiltration without clear spatial orientation of cells (**A**) and an iBALT cluster with a high density of epithelioid cells (**B**) and a large focus of necrosis (**C**) in the lung of an untreated infected mouse. Organizing stage necrosis foci (**D**), foci of specific infiltration (**E**), and an iBALT aggregate with small numbers of epithelioid cells (**F**) in the lungs of mice treated with anti-TB drugs alone for 4 months. A small area of specific infiltration (**G**) and a lymphoid peribronchial iBALT cluster (**H**) in the lungs of mice immunized three times with TB/FLU-06E 2 weeks after the start of tuberculosis therapy. Hematoxylin and eosin staining. Magnification ×300 (**A**–**E**,**G**) or ×600 (**F**,**H**). The black arrows indicate the described histological changes.

In the untreated infected control (CI), the survival rate was 30% (Figure 4B), and the bacterial load was 5.43 lg CFU 140 days post-infection (Figure 4C). Histologically, all animals developed confluent foci of infiltration in the lungs with no clear spatial orientation of the cells (Figure 5A), large foci of necrosis with recent infiltrative changes and no signs of fibrosis (Figure 5C), and iBALT clusters with extensive inclusions of epithelioid cells (Figure 5B). Four months of treatment with anti-TB drugs chosen according to the sensitivity of the *M. tuberculosis* strain (AETppBq) resulted in a 1.33 lg CFU decrease in the bacterial load in the lungs, and the survival rate increased to 90%. The treatment also reduced the area of specific inflammation foci in the lungs. In 50% of the cases, these foci retained their confluent character, while in the other 50%, they were represented by small areas of infiltration with a large number of foamy macrophages (Figure 5E). In one of the lung sections of this group, large foci of necrosis were recorded at the stage of organization, which suggests progressive lung damage (Figure 5D). At the same time, only small accumulations of epithelioid cells within iBALT clusters were observed in the foci of infiltration (Figure 5F), and large perivascular lymphohistiocytic infiltrates were detected.

Finally, we compared two TB/FLU-06E immunotherapy regimens: double versus triple immunization. The regimens involved 4-week intervals between vaccinations, with the first administration occurring 2 weeks after the start of causal treatment. The results indicated that triple immunization significantly enhances the efficacy of TB/FLU-06E therapy by improving pulmonary clearance of *M. tuberculosis* (0.93 lg CFU, *p* < 0.0074; Figure 4C). In contrast, double vaccination reduced bacterial elimination by only 0.33 lg CFU. In the lungs of triple-immunized mice, specific lesions were the least common among all groups of treated animals, and only small areas of infiltration were observed (Figure 5G). Moreover, unlike the treatment control (AETppBq), their cellular composition included neither foamy macrophages nor alternative inflammatory components such as neutrophils or destructive foci. After triple immunization with TB/FLU-06E, the iBALT clusters in the infiltrates were smaller and consisted solely of lymphocytes, whereas the treatment control developed iBALT clusters with epithelioid cells (Figure 5H). Mice treated three times with TB/FLU-06E also had increased peribronchial lymphohistiocytic infiltration, which was not detected in the treatment control (*p* = 0.005).

**Figure 6:**
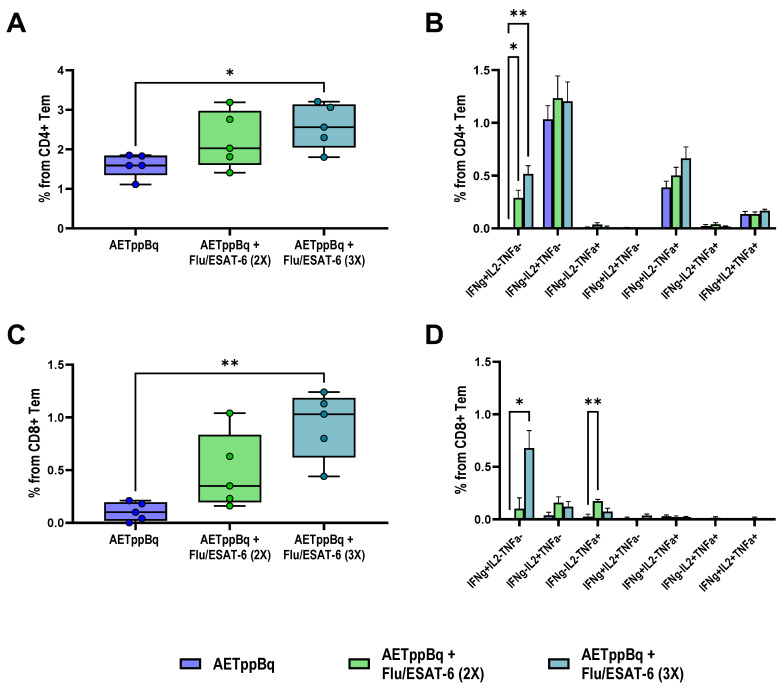
Systemic TB-specific immune response following DR-TB treatment. Proportions of cytokine-producing CD4+ (**A**,**B**) and CD8+ (**C**,**D**) effector memory T cells in spleens of C57BL/6 mice infected with *M. tuberculosis* (Beijing family) after 4 months of AETppBq therapy with TB/FLU-06E (intranasally, double (2×) or triple (3×) administration). The frequencies of IFN-γ-, TNF-α-, and IL-2-producing CD4+ and CD8+ Tem cells were measured by flow cytometry (ICS). Background signal from cells stimulated only with the medium has been subtracted. The data were considered statistically significant when *p* < 0.05, as determined by one-way or two-way ANOVA with Tukey’s multiple comparisons test (* *p* < 0.05, ** *p* < 0.01).

The advantage of the triple TB/FLU-06E immunization was also evident when assessing the formation of cytokine-producing Tem cells. Significantly higher counts of cytokine-producing antigen-specific CD4+ (*p* = 0.0334) and CD8+ (*p* = 0.0062) Tem were recorded in splenocyte cultures from triple-immunized mice compared with mice treated only with anti-TB drugs (Figure 6A,C).

## 4. Discussion

Treating patients with multidrug-resistant and extensively drug-resistant tuberculosis is challenging due to the complexity, high costs, and low efficiency of treatment, as resistance usually occurs against the most important and effective anti-TB drugs [25]. The treatment efficacy for patients with MDR or XDR tuberculosis remains low worldwide, with success rates of approximately 50% and 30%, respectively [3,26].

Our study found a pronounced delay in the regression of DR tuberculosis in mice infected with *M. tuberculosis* Beijing, even with a four-month course of therapy using anti-TB drugs chosen based on the drug sensitivity profile of the mycobacteria. When the virulent strain *M. tuberculosis* H37Rv was used to model drug-sensitive infection, a 2.5-month course of specific chemotherapy (HR) resulted in a 3.38 lg reduction in the bacterial load compared with untreated animals. In contrast, in mice infected with *M. tuberculosis* Beijing, 4 months of treatment with anti-TB drugs reduced the bacterial load in the lungs by only 1.33 lg CFU.

A possible solution to the problem of low treatment efficacy in DR infection is to modulate the microorganism’s immune response, which may be compromised due to both tuberculosis infection and anti-TB treatment [6]. The negative influence of treatment is supported by a significant relapse rate observed in treated patients with DR tuberculosis, with 81% of cases linked to endogenous reactivation [27].

In this study, we used TB/FLU-06E, a recombinant influenza vaccine vector expressing ESAT-6, as an immunotherapeutic agent. It has previously demonstrated efficacy in prophylactic and therapeutic vaccination against experimental tuberculosis by inducing a pronounced antigen-specific systemic Th1-cell response and leveraging the immunoadjuvant properties of the influenza vector itself [14,15]. Of note, in our previous studies, we observed that both wt influenza virus and empty influenza vector showed a moderate nonspecific protective effect during Mtb infection, but this effect was significantly lower than when vector carrying ESAT-6 was used [14,28].

Included in the complex anti-TB therapy, TB/FLU-06E significantly increased the efficacy of treatment of both DS and DR tuberculosis. Thus, when *M. tuberculosis* H37Rv was inoculated, intranasal immunization of mice with TB/FLU-06E in the optimal regime reduced *M. tuberculosis* shedding from lungs by 1.83 lg CFU compared with mice treated with anti-TB drugs alone. For DR tuberculosis, shedding decreased by 0.93 lg CFU. In addition to increasing the clearance of *M. tuberculosis* from the lungs in both forms of infection, the TB/FLU-06E immunotherapy reduced specific inflammation and the severity of exudative and necrotic lung damage in DR tuberculosis. Furthermore, it stimulated the lymphoid component of the cellular immune response in the lungs. Within foci of specific granulomatosis, this was characterized by changes in the cellular composition of lymphoid clusters and an increase in periarterial and peribronchial lymphohistiocytic infiltration. These lymphoid clusters are part of inducible broncho-associated lymphoid tissue (iBALT). They are tertiary lymphoid organs induced in the lungs in response to inflammatory stimuli caused by unresolved infection, such as tuberculosis [29,30]. iBALT is typically observed near the bronchi, but it can also be localized in the perivascular or interstitial regions of the lungs during tuberculosis infection, regulating the host’s adaptive immune response to *M. tuberculosis* [31,32,33]. The development of iBALT requires an inducible inflammatory trigger, with T-helpers—particularly IL-17-secreting Th lymphocytes—and the effector cytokines they produce acting as key initiators [30,34,35]. Overall, the formation of iBALT in tuberculosis infection suggests an effective immune response and favorable disease outcome [30,36,37].

In both DS and DR tuberculosis models, TB/FLU-06E treatment increased the occurrence of large periarterial and peribronchial lymphohistiocytic infiltrates and resulted in a marked rearrangement of the cellular composition of iBALT lymphoid clusters in the areas of specific granulomatosis foci. These clusters primarily consisted of lymphoid cells in TB/FLU-06E-treated mice, whereas in mice treated with anti-TB drugs alone, the clusters contained foci of epithelioid cells.

The differences stem from the regression of specific lesions due to the experimental therapy. Untreated mice infected with *M. tuberculosis* H37Rv or *M. tuberculosis* Beijing showed widespread infiltration in both tuberculosis models, developing foci of necrosis (in DR tuberculosis) combined with large iBALT aggregates accumulating epithelioid cells. On the other hand, mice treated with antibiotic therapy alone showed smaller iBALT clusters containing fewer epithelioid cells and a reduced area of inflammation. The presence of epithelioid cells in the iBALT clusters, albeit in small numbers, suggests that the immune response was not sufficiently effective, allowing the intracellular reservoir of infection to persist [32]. The ineffectiveness of even a long, four-month course of specific chemotherapy in DR tuberculosis is further supported by histological evidence, as we observed a large number of foamy macrophages within the infiltration foci. *M. tuberculosis* is known to affect acyl-CoA cholesterol acyltransferase (ACAT), thereby disrupting lipid metabolism and facilitating the fusion of *M. tuberculosis*-containing phagosomes with lipid bodies. This allows *M. tuberculosis* to switch into a dormant phenotype, protecting it from the bactericidal effects of the respiratory burst [38].

In contrast to the control groups, TB/FLU-06E-treated mice did not exhibit epithelioid cells in the iBALT clusters or foamy macrophages in the infiltrates, which confirmed the efficacy of immunotherapy in terms of clearing the lungs of the pathogen. The presence of iBALT lymphoid cells and lymphohistiocytic infiltrates in these mice may indicate the activation of the lymphoid component of the local pulmonary immunity, which occurs when tuberculosis infection activity decreases due to immunotherapy [11,37,39].

The cytokine-producing activity of specific memory T cells is often considered a correlate of protection, both in assessing the effect of prophylactic vaccination and in experimental and clinical studies of the efficacy of tuberculosis therapy. Phenotypic and functional profiles of antigen-specific memory T cells are used for the differential diagnosis of latent and active tuberculosis [40,41]. Patients with active tuberculosis have been shown to have a higher proportion of central IFN-γ+TNF-α+ CD4+ T cells and a lower proportion of CD8+ effector T lymphocytes [42]. Active tuberculosis is also associated with a decrease in polyfunctional and IL-2+ T cells and an increase in TNF-α+ CD4+ and CD8+ memory T cells [43]. Clinical studies have demonstrated certain shifts in cytokine-producing memory cell populations after anti-TB therapy: the successful treatment of patients with active tuberculosis led to an increase in the proportion of CD8+ Tem cells producing IFN-γ and IFN-γ plus IL-2 among peripheral blood mononuclear cells (PBMCs) [42,44].

In experimental studies, the population of CD8+ Tem cells was observed to decrease during the long-term persistence of *M. tuberculosis* in mice [6] and as a result of the immunosuppressive effect of anti-TB drugs [10].

According to our results, the therapeutic effect of optimal TB/FLU-06E vaccine therapy regimens was accompanied by a simultaneous increase in cytokine-producing activity of both CD4+ and CD8+ Tem cells. In the DS tuberculosis model, double immunization with TB/FLU-06E at the start of the HR therapy resulted in a significantly higher level of total spleen-derived cytokine-producing CD4+ and CD8+ Tem cells (CD44+ CD62L-), with a markedly higher proportion of IFN-γ and IFN-γ,TNF-α producing cells. The therapeutic effect of triple immunization with TB/FLU-06E in the DR tuberculosis model was accompanied by an increase in the counts of cytokine-producing CD4+ and CD8+ Tem cells predominately represented by IFN-γ-producing lymphocytes. The activation of *M. tuberculosis*-specific CD4+/CD8+ Tem cells in our study, accompanied by a lower bacterial load and reduced prevalence of specific lung lesions, appeared to be a consequence of immunotherapy. The TB/FLU-06E immunotherapy impacts the persistence of TB antigens during tuberculosis infection triggering the dysfunction of CD4+ and CD8+ Tem cells [45,46]. Some differences in the effects of TB/FLU-06E therapy on the formation of Tem cells between the models with different *M. tuberculosis* susceptibility may also be related to the antigenic load. In particular, we observed a higher residual pulmonary bacterial load in DR tuberculosis, where the efficacy of a four-month anti-TB therapy regimen based on the drug sensitivity profile was significantly lower than that of a two-month course of HR in DS infection.

An additional benefit of the TB/FLU-06E vaccine therapy in both tuberculosis models was the activation of the CD8+ T-cell response. The role of CD8+ T cells in protective immunity during tuberculosis [46,47] and the development of latent infection [48] is currently under active investigation. The formation of CD8+ memory cells has been suggested as one of the criteria for assessing the efficacy of tuberculosis treatment. Thus, some authors argue that the lack of effector CD8+ T lymphocytes underlies the insufficient protection in patients who have developed active tuberculosis [42]. CD8+ T cells are believed to primarily contribute to the fight against *M. tuberculosis* due to their ability to generate a cytotoxic response through inducing cell apoptosis, as well as releasing perforins, granzymes, and granulysin (for humans). CD8+ T cells can also produce pro-inflammatory cytokines IFN-γ, TNF-α, IL-2, and IL-17, often at frequencies similar to CD4+ T cells [43,49]. However, they cannot compensate for the lack of CD4+ T cells, a subpopulation critical for immune defense in tuberculosis [48,50,51]. Furthermore, in addition to their direct functions in the response to infection, CD8+ T cells also play an important role in organizing optimal CD4+ T-cell function in granulomas [43]. Yet, the exact role of CD8+ T cells in the initiation, progression, and outcome of tuberculosis infection remains to be elucidated.

In conclusion, here we have shown that therapeutic immunization with the TB/FLU-06E vaccine significantly increases the efficacy of anti-TB therapy in both DS and DR experimental tuberculosis models. The vaccination can reduce the duration of antibiotic therapy, which is particularly important in DR tuberculosis characterized by delayed resolution of specific inflammation in the lungs.

## Data Availability

The data presented in this study are available on reasonable request from the corresponding author.

## Supplementary Materials

The following supporting information can be downloaded at https://www.mdpi.com/article/10.3390/pharmaceutics16070857/s1: Figure S1: Gating strategy used to identify cytokine-producing Tem cells. Cell doublets were eliminated using FSC-A/FSC-H light scattering. The live single-cell population was determined based on the FSCA/SSC-A light scattering and binding of the Zombie Red dye; Figure S2: Representative plots (merged data) demonstrate BCG-induced cytokine production in CD4+ Tem cells derived from spleens of C57BL/6 mice infected with *M. tuberculosis* H37Rv, after 2.5 months of HR therapy with TB/FLU-06E (intranasally, twice); Figure S3: Representative plots (merged data) demonstrate BCG-induced cytokine production in CD8+ Tem cells derived from spleens of C57BL/6 mice infected with *M. tuberculosis* H37Rv, after 2.5 months of HR therapy with TB/FLU-06E (intranasally, twice); Figure S4: Representative plots (merged data) demonstrate BCG-induced cytokine production in CD4+ Tem cells derived from spleens of C57BL/6 mice infected with *M. tuberculosis* (Beijing family) after 4 months of AETppBq therapy with TB/FLU-06E (intranasally, double (2×) or triple (3×) administration); Figure S5: Representative plots (merged data) demonstrate BCG-induced cytokine production in CD8 Tem cells derived from spleens of C57BL/6 mice infected with *M. tuberculosis* (Beijing family) after 4 months of AETppBq therapy with TB/FLU-06E (intranasally, double (2×) or triple (3×) administration); Table S1: Experimental design: TB/FLU-06E therapy in a drug-susceptible tuberculosis model (n = 73); Table S2: Experimental design: TB/FLU-06E therapy in a drug-resistant tuberculosis model (n = 73).
