# Supplementary material for: TB/FLU-06E Influenza Vector-Based Vaccine in the Complex Therapy of Drug-Susceptible and Drug-Resistant Experimental Tuberculosis
Source: Pharmaceutics. 2024 Jun 25;16(7):857. doi: 10.3390/pharmaceutics16070857 (PMC11279844; doi:10.3390/pharmaceutics16070857)
Supplement: Supplementary file 1 [file pharmaceutics-16-00857-s001.zip › pharmaceutics-3014119-supplementary.pdf]

pharmaceutics

Supplementary

Table S1. Experimental design: TB/FLU-06E therapy in a drug-susceptible tuberculosis model (n=73).

Group	Number of animals	TB infection control	Survival	Bacterial growth, histology (lungs)	ICS (spleens)[1]
CI	25	9/25	10/16	6/6	
HR	16	-	10/16	6/6	
HR+TB/FLU-06E	16	-	10/16	6/6	
HR+TB/FLU-06E (1m)	16	-	10/16	6/6	

[1] ICS was performed in 5 animals per group. The data for the CI group are not presented. Due to the strong influence of HR therapy on the immune status, only HR-treated groups were compared.

Table S2. Experimental design: TB/FLU-06E therapy in a drug-resistant tuberculosis model (n=73).

Group	Number of animals	TB infection control	Survival	Bacterial growth, histology (lungs)	ICS (spleens)[1]
CI	25	9/25	10/16	6/6 [2,3]	
AETppBq	16	-	10/16	6/6 [2,3],	
AETppBq + TB/FLU-06E (2x)	16	-	10/16	6/6 [3]	
AETppBq + TB/FLU-06E (3x)	16	-	10/16	6/6	

[1] ICS was performed in 5 animals per group The data for the CI group are not presented. Due to the strong influence of AETppBq therapy on the immune status, only AETppBq-treated groups were compared.

[2] By d140, 5 animals had survived

[3] Bacterial growth results available for 4 animals only

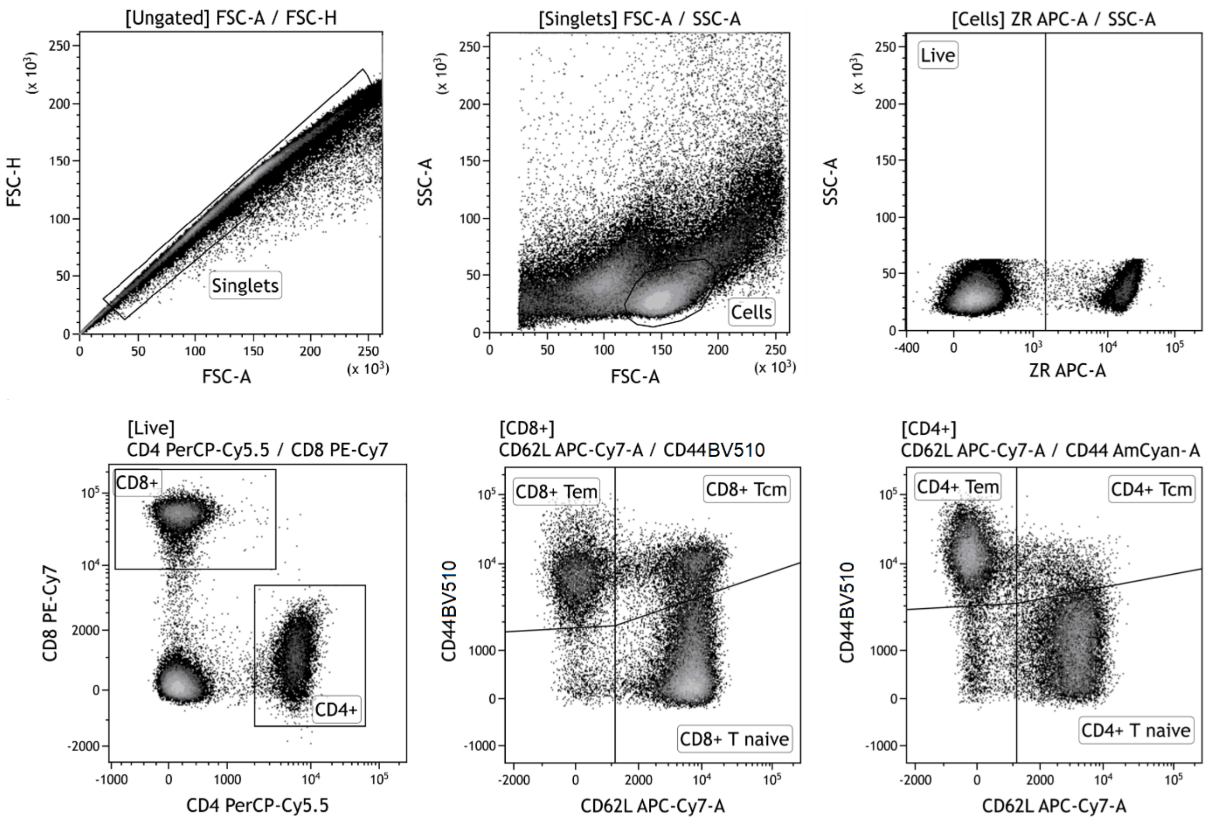

Figure S1. Gating strategy used to identify cytokine-producing Tem cells. Cell doublets were eliminated using FSC-A/FSC-H light scattering. The live single-cell population was determined based on the FSCA/SSC-A light scattering and binding of the Zombie Red dye. T-helper cells were identified as CD4+, CTLs as CD8+. Using CD44 and CD62L markers, CD4+ and CD8+ cells were further subdivided into naïve (CD44-CD62L+), Tcm (CD44+CD62L+), and Tem (CD44+CD62L-) cells. IFN-γ, IL-2, and TNF-α responses were evaluated in Tem cells.

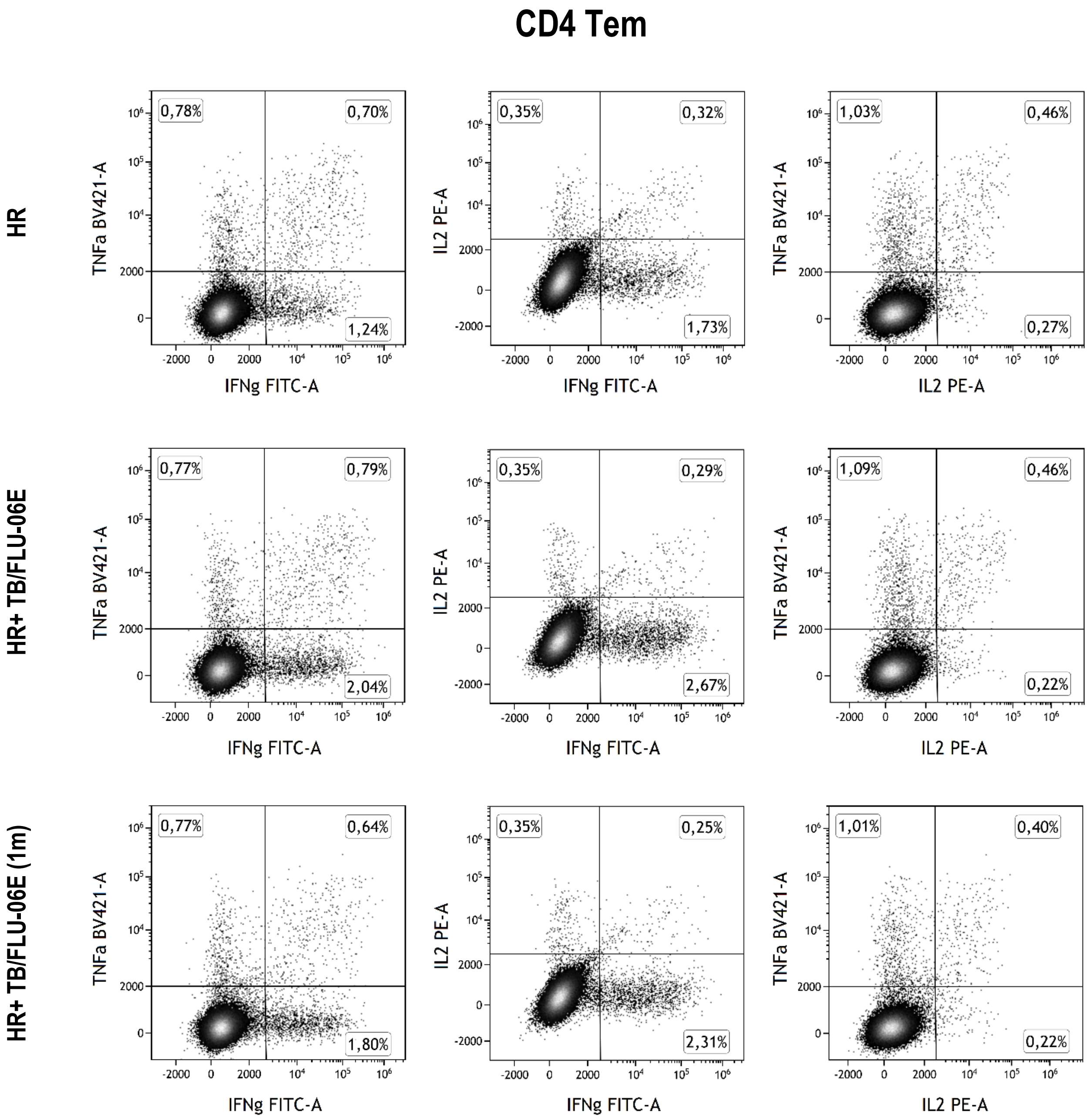

Figure S2. Representative plots (merged data) demonstrate BCG-induced cytokine production in CD4+ Tem cells derived from spleens of C57BL/6 mice infected with *M. tuberculosis* H37Rv, after 2.5 months of HR therapy with TB/FLU-06E (intranasally, twice).

pharmaceutics

MDPI

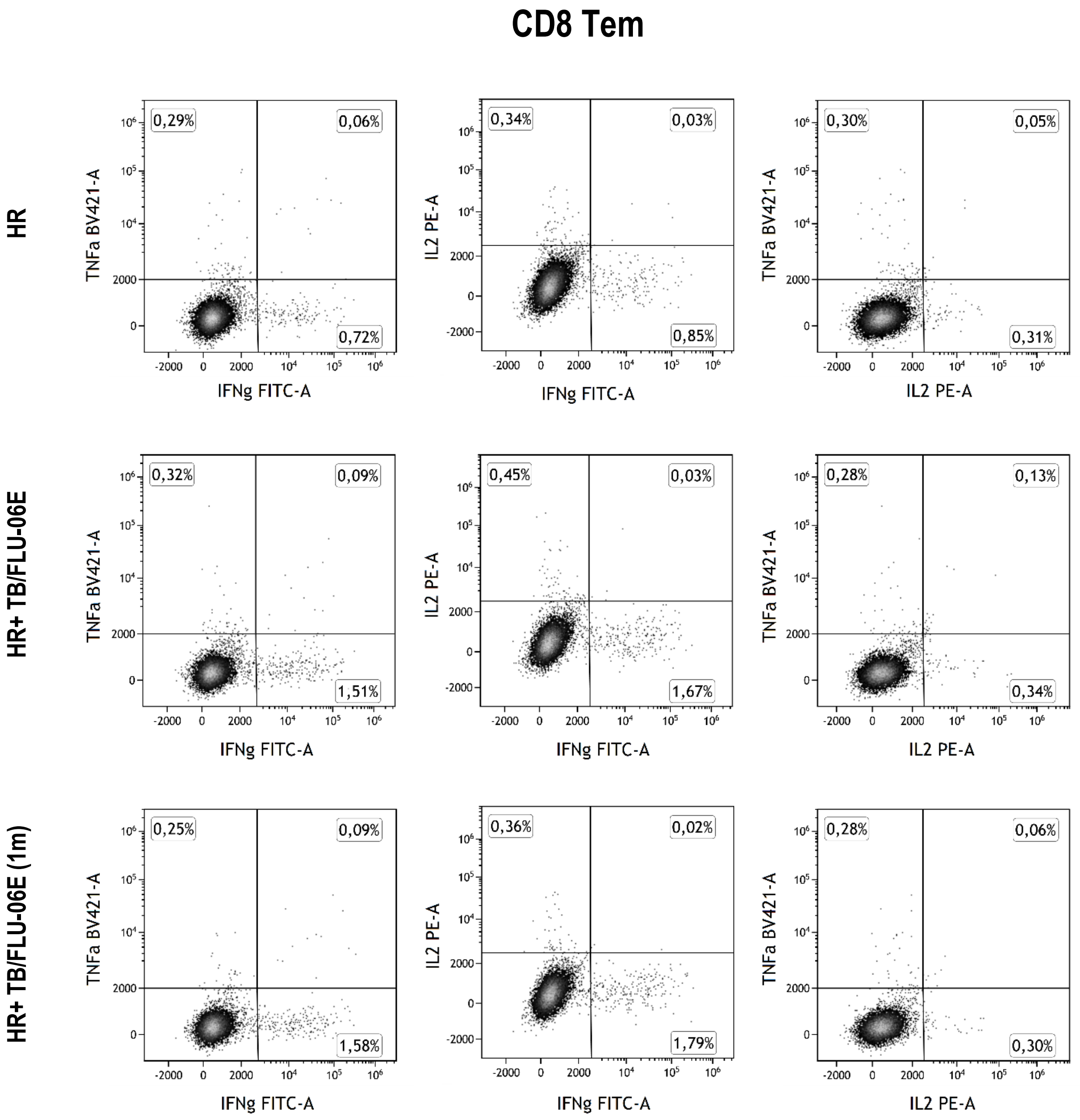

Figure S3. Representative plots (merged data) demonstrate BCG-induced cytokine production in CD8+ Tem cells derived from spleens of C57BL/6 mice infected with *M. tuberculosis* H37Rv, after 2.5 months of HR therapy with TB/FLU-06E (intranasally, twice).

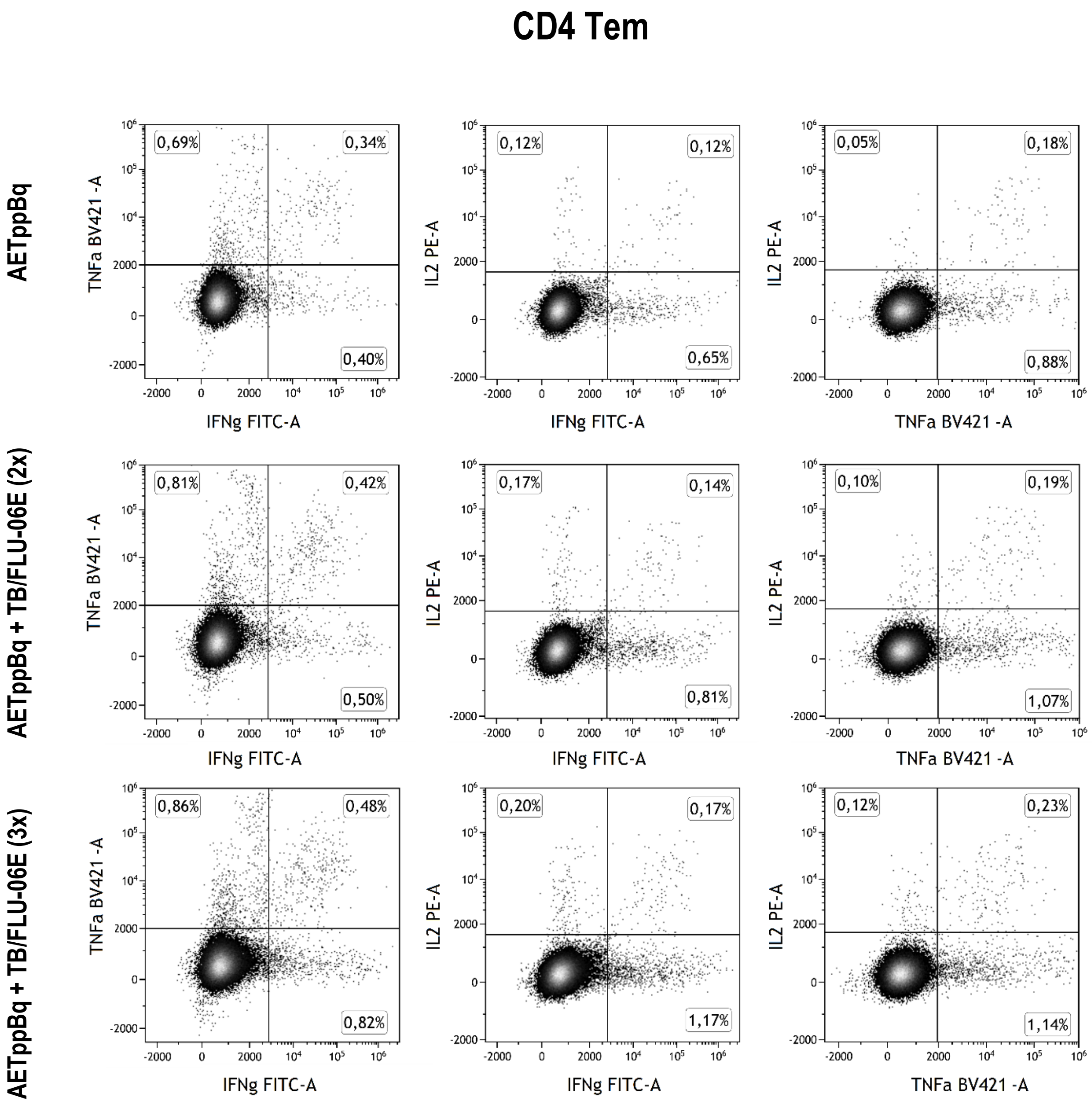

Figure S4. Representative plots (merged data) demonstrate BCG-induced cytokine production in CD4+ Tem cells derived from spleens of C57BL/6 mice infected with M. tuberculosis (Beijing family) after 4 months of AETppBq therapy with TB/FLU-06E (intranasally, double (2X) or triple (3X) administration).

CD8 Tem

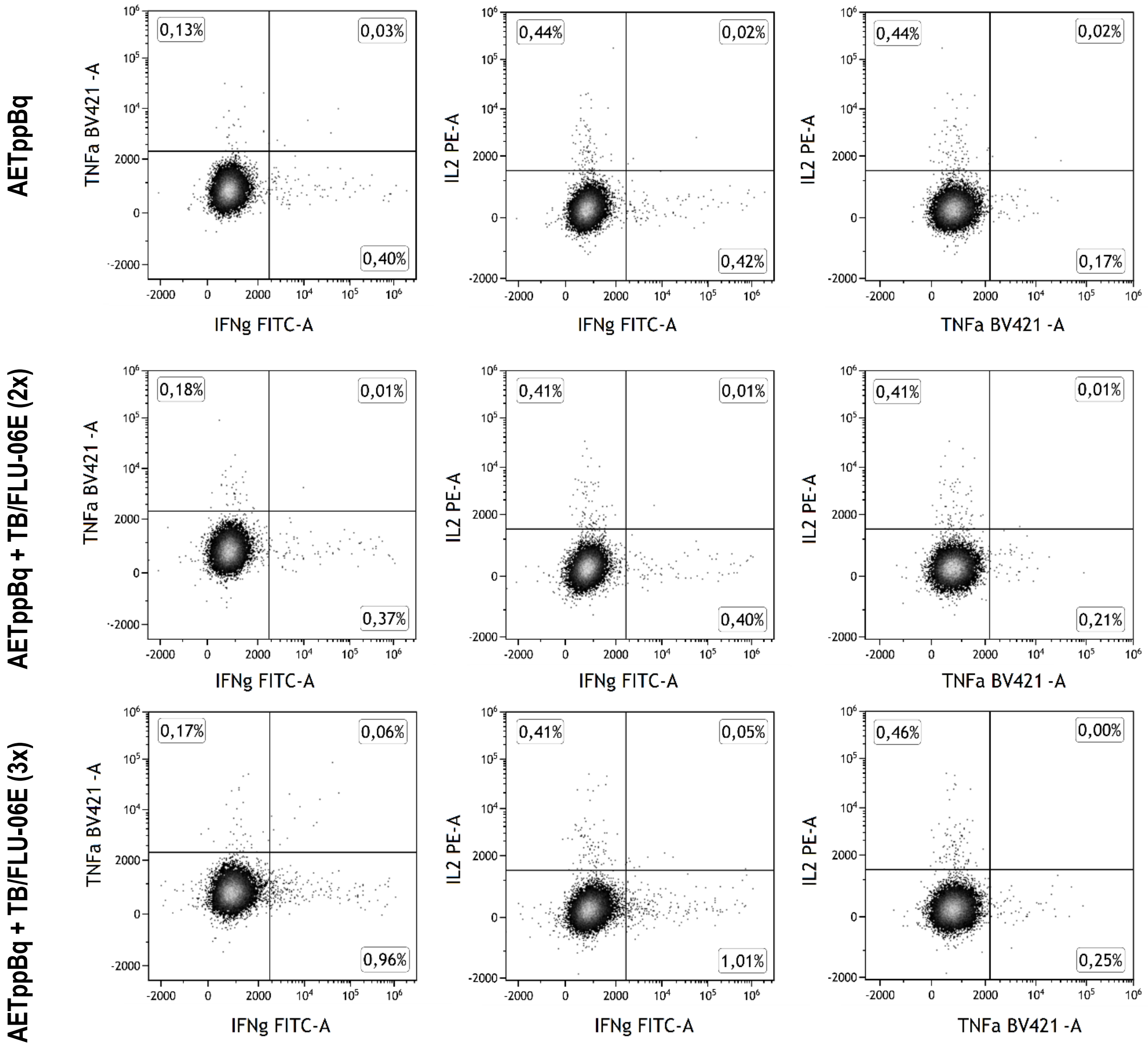

Figure S5. Representative plots (merged data) demonstrate BCG-induced cytokine production in CD8+ Tem cells derived from spleens of C57BL/6 mice infected with M. tuberculosis (Beijing family) after 4 months of AETppBq therapy with TB/FLU-06E (intranasally, double (2X) or triple (3X) administration).
